# Intracellular Transport Routes for MHC I and Their Relevance for Antigen Cross-Presentation

**DOI:** 10.3389/fimmu.2015.00335

**Published:** 2015-07-02

**Authors:** Aimé Cézaire Adiko, Joel Babdor, Enric Gutiérrez-Martínez, Pierre Guermonprez, Loredana Saveanu

**Affiliations:** ^1^INSERM U1149, Faculté Bichat Medical School, ELR8252 CNRS, Center for Research on Inflammation, Paris, France; ^2^Université Paris Diderot, Sorbonne Paris Cité, Paris, France; ^3^INSERM UMR 1163, Laboratory of Cellular and Molecular Mechanisms of Hematological Disorders and Therapeutic Implications, Paris, France; ^4^Université Paris Descartes, Sorbonne Paris Cité, Paris, France; ^5^Imagine Institute, Paris, France; ^6^Laboratory of Phagocyte Immunobiology, Peter Gorer Department of Immunobiology, King’s College London, London, UK

**Keywords:** MHC class I molecules, cross-presentation, endosomal recycling compartment, tubular endosomes, dendritic cells

## Abstract

Cross-presentation, in which exogenous antigens are presented via MHC I complexes, is involved both in the generation of anti-infectious and anti-tumoral cytotoxic CD8^+^ T cells and in the maintenance of immune tolerance. While cross-presentation was described almost four decades ago and while it is now established that some dendritic cell (DC) subsets are better than others in processing and cross-presenting internalized antigens, the involved molecular mechanisms remain only partially understood. Some of the least explored molecular mechanisms in cross-presentation concern the origin of cross-presenting MHC I molecules and the cellular compartments where antigenic peptide loading occurs. This review focuses on MHC I molecules and their intracellular trafficking. We discuss the source of cross-presenting MHC I in DCs as well as the role of the endocytic pathway in their recycling from the cell surface. Next, we describe the importance of the TAP peptide transporter for delivering peptides to MHC I during cross-presentation. Finally, we highlight the impact of innate immunity mechanisms on specific antigen cross-presentation mechanisms in which TLR activation modulates MHC I trafficking and TAP localization.

## A Short History of Major Histocompatibility Complex (MHC) Identification

The MHC is a key component of the immune system that allows T lymphocytes to specifically detect foreign antigens in the context of a self-identification system. The discovery of MHC genes, as genetic loci controlling the immune response, originated from organ transplantation experiments and was performed between 1940 and 1970 by George D. Snell, Jean Dausset, and Baruj Bernacerraf, rewarded by the Nobel Prize in 1980 for these discoveries (1). In 1975, while studying the immune response of mice to viruses, Doherty and Zinkernagel demonstrated the relevance of T cell restriction by MHC. They showed that T cells were able to recognize viral peptides only if these peptides are bound to MHC and highlighted for the first time the phenomenon of “MHC restriction” (2). These major discoveries have also been rewarded, in 1996, by a Nobel Prize. The principle of parallel recognition of both self and non-self molecules was the basis for our current understanding of the specificity of the cellular immune response. In parallel, the study of antibody response in inbred congenic animal models led to the discovery of MHC II and established the distinction between MHC I and MHC II [for a comprehensive review on MHC II discovery, see Ref. (3)].

Further research on MHC antigen presentation established that MHC I binds antigenic peptides derived from endogenous antigens and present them to CD8^+^ T cells, while MHC II binds antigenic peptides from exogenous antigens and present them to CD4^+^ T cells. This dichotomy is not absolute and exceptions exist at several levels. The most recent, and also one of the most surprising exceptions, is the discovery of an efficient CD8^+^ T cytotoxic response that is MHC II restricted, in the case of monkey vaccination with a cytomegalovirus vector expressing antigens from the simian immunodeficiency virus (4).

While the concept that CD8^+^ T cells are exclusively MHC I restricted has been re-evaluated only recently, the idea that MHC I present exclusively endogenous antigens has been reconsidered nearly 40 years ago, when Bevan discovered the phenomenon of CD8^+^ T cells cross-priming (5). He showed that minor histocompatibility antigens from transplanted cells can elicit a cytotoxic T cell response restricted by host MHC I molecules, which revealed that the source of antigenic peptides presented by MHC I might also be an exogenous antigen, and not only an antigen synthesized inside the presenting cell.

## Contribution of Cross-Presentation to Immunity and Self-Tolerance

The presentation of exogenous antigens by MHC I has been named cross-presentation. Following the discovery of CD8^+^ T cell cross-priming in transplant rejection (5), additional studies demonstrated that cross-presentation also occurs in the case of several cell-associated antigens, such as self-antigens (6), viral antigens (7), or antigens derived from tumoral cells (8). In addition to these typical cell-associated antigens, an important source of cross-presented material is the proteins synthesized by intracellular pathogens, such as *Listeria* (9), *Plasmodium* (10), or *Leishmania* (11). In these infections, the CD8^+^ T cells response contributes to protective immunity [reviewed in Ref. (12)].

Early during the study of cross-presentation, it was discovered that this process can lead not only to CD8^+^ T cell activation (T cell cross-priming) but also to T cell tolerance by either T cell anergy or T cell deletion [Cross-tolerance (13)]. How exactly cross-presentation influences the outcome of T cell response is a topic of particular interest that was extensively studied in the last two decade (14). Early after the discovery of tolerogenic abilities of cross-presentation, it has been supposed that there are several types of antigen cross-presenting cells, some which induce predominantly T cell activation and others that promote T cell tolerance. Alternatively, it has been proposed that the same antigen cross-presenting cell can be switched from a tolerogenic to an immunogenic status according to the nature of the engulfed antigenic material. As a result of significant efforts of several laboratories in the field, it became evident that in dendritic cells (DCs), it is the second scenario that reflects the plasticity of the cross-presentation process to orient the final outcome of the immune response toward cross-priming or cross-tolerance.

## Overview of Dendritic Cell Populations

Dendritic cells are by far the most effective cross-presenting cells in both homeostatic and inflammatory conditions (15). As widely demonstrated by studies done both in humans and mice, DCs are a very heterogeneous cellular population (16–18). In a homeostatic situation and without mentioning the skin and mucosal DCs, the murine DC system in lymphoid organs is composed of conventional DCs (CD8^+^ and CD8^−^) and plasmacytoïd DCs (pDCs). In humans, DCs found in the blood, spleen, and lymph nodes are classified as pDCs, BDCA1^+^ DCs, and BDCA3^+^ DCs. Based on gene-expression profiles, their function, and morphology, BDCA3^+^ DCs are considered to be the equivalent of murine CD8^+^ DCs and BDCA1^+^, the equivalent of murine CD8^−^ DCs (19–22). Conventional DCs require for their differentiation the cytokine FMS-like tyrosine kinase 3 ligand (FLT3L) and additional sub-type specific transcription factors, describing differentiation pathways. A unified ontogeny-based nomenclature of mouse and human DCs has been recently proposed for conventional DCs, which are grouped in three main sub-types: classical type 1 DCs (cDC1), classical type 2 DCs (cDC2), and pDCs (23). The cDC1 sub-type is dependent on the transcription factor Baft3 and includes murine CD8^+^ DCs, murine CD103^+^ DCs, and human BDCA3^+^ DCs. The cDC2 sub-type requires the transcription factor IRF4 and includes murine CD4^+^ DCs and human BDCA1^+^ DCs, while the differentiation of pDCs requires E2-2 transcription factor.

If in the murine DCs system, there is a degree of specialization of DC subsets for cross-presentation, with splenic CD8^+^ DCs and tissue resident CD103^+^ DCs being recognized as the most efficient cross-presenting subsets (16), in humans, all DC subsets display similar levels of ability for cross-presentation *ex vivo* (24). In addition to the DC populations found in homeostatic conditions, in inflammatory conditions, in both humans and mice, the monocytes can differentiate in several “DC-like” cellular populations known as monocyte-derived DCs. Considering their ontogeny, which is different from that of conventional DCs, the new name “monocyte-derived cells” has been recently proposed for highly heterogeneous cellular populations that arise from monocytes in inflammatory conditions (23). Although sub-types of monocyte-derived cells are able to capture antigens *in vivo* and to cross present them via MHC I *in vitro* (25), their role *in vivo* for the priming of CD8^+^ T cells remains to be elucidated. The study of these subclasses will become possible as soon as the transcription factors required for their differentiation will be identified, to allow targeted depletion of specific monocyte-derived cells.

Some of DC subsets induce cross-tolerance in steady-state conditions (26–28) and cross-priming when the antigenic material contains a “danger signal” that will stimulate one of the pattern recognition receptors (PRRs) of DCs, such as toll-like-receptors (TLRs), NOD-like receptors (NLRs), or RIG-I-Like Receptors (RLRs) (29). Thus, phagocytosis of apoptotic cells, which lack “danger” signals and do not activate the PRRs, triggers an anti-inflammatory response (30) and cross-tolerance. In contrast, phagocytosis of pathogens, which activates the PRRs, triggers an inflammatory response and cross-priming. Signaling by PRRs thus leads to profound modifications of DCs, which become able to provide signals that are mandatory for cross-priming. These signals include recognition of peptide MHC complex (pMHC I) by the T cell receptor (TCR), followed by the interaction of DC’s co-stimulatory molecules CD80, CD86 with CD28 on T cells and polarized secretion of pro-inflammatory cytokines (IL-12) toward the immunological synapse (31).

In conclusion, a critical feature of DCs is their ability to coordinate antigen presentation, including MHC I cross-presentation, with the physiological context, like the presence of inflammatory cytokines or danger signals from pathogens. Among the complex cell-intrinsic mechanisms that allow this coordination, the regulation of MHC I trafficking during cross-presentation is a critical factor that will be discussed in detail in this review.

## Accessory Cells Involved in MHC I Cross-Presentation

In addition to DCs, several cell types were shown to be able to perform cross-presentation in particular situations. These include macrophages (32, 33), liver sinusoidal endothelial cells (LSECs) (34), endothelial cells (35), γδT cells (36), mast cells (37), and B cells (38). Although unusual, these events of cross-presentation might be relevant for CD8^+^ T cell priming against tumor antigens (33) or poorly vascularized transplanted organs (35). These particular antigen cross-presentation events are usually prompted by inflammatory conditions; with the exception of LSECs-mediated cross-presentation that occurs under homeostatic conditions and leads to CD8^+^ T cell tolerance (39). Interestingly, even in the presence of inflammatory triggers, such as TLR4 ligands, the antigen cross-presentation by LSECs remains tolerogenic (40), which indicates that cellular biology of antigen processing and cross-presentation in LSECs should be different from how it occurs in DCs.

## Molecular Players Involved in MHC I Assembly

**Figure 1:**
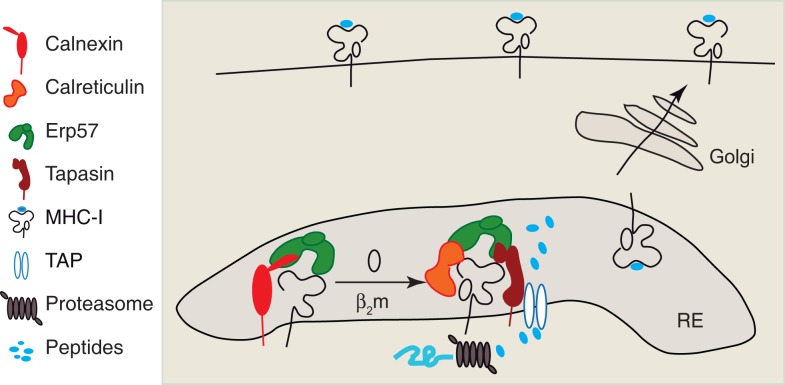
**Intracellular assembly of MHC I peptide complex**. The newly synthetized heavy chain of MHC I interacts initially with Calnexin and associated thiol oxidoreductase ERp57, and is retained in the ER, where the HC associates with β_2_m. The HC–β_2_m complex interacts with the peptide-loading complex (PLC) formed by Calreticulin, ERp57, Tapasin, and the peptide transporter TAP. Antigenic peptide precursors from cytosol are transported by TAP in the ER lumen, where they are processed by ER trimming aminopeptidases (ERAPs). Once the optimal epitope binds the HC–β_2_m complex, the MHC I is released from the PLC, is packaged into COP-II vesicles, and undergoes the anterograde transport on the secretory pathway. MHC I lacks obvious signal motifs for anterograde transport and the molecular events leading to MHC I packaging into COP-II vesicles remain unknown.

MHC I is a heterodimeric complex containing a glycosylated transmembrane protein called heavy chain (HC), a small soluble protein, the beta-2 microglobulin (β_2_m) and a peptide ligand that is 8–10 aminoacids in length. In all nucleated cells of the body that can present endogenous antigens via MHC I, newly synthesized heavy chains (HC) are translocated in the endoplasmic reticulum (ER), where the HC assembly with β_2_m and peptide binding occurs. Like many other cells, DCs are also capable to present antigens that are expressed in their cytosol after endogenous protein synthesis from viral or endogenous mRNAs. During endogenous MHC I presentation, the antigenic peptides are produced in the cytosol by the concerted action of several cytosolic proteases, among which the proteasome contributes a major fraction (41). The ABC peptide transporter TAP transports these antigenic peptides from the cytosol in the ER, where they can bind the newly synthesized HC. MHC I assembly in the ER is a complex process in which a series of dedicated chaperons assist the correct folding of HC. An overview of molecular events leading to MHC I assembly and peptide loading in the ER, comprehensively reviewed in Ref. (42–44) is depicted in Figure 1.

After the binding of a high affinity peptide, MHC I complex may be exported outside ERGIC to reach the cell membrane. Both peptide-loaded and peptide free human MHC I can be visualized by immunoblot and fluorescence microscopy using conformational antibodies (e.g., W6.32 that recognizes peptide-loaded MHC I and HC-10 that detects free HC). It was thus possible to visualize peptide-free HC outside the ER, in the ERGIC, and cis-Golgi. Besides peptide-receptive MHC I (45), other components of MHC I peptide-loading complex, such as functional TAP (46) and calreticulin or tapasin (47), have been detected in ERGIC, suggesting that peptide loading might occur outside ER, in the early secretory pathway. The molecular events involved in the exit of both pMHC and free HC of MHC I from the ER toward the Golgi stacks, remain unknown. The cytosolic tail of HC does not contain any previously described ER–Golgi trafficking signals (48), which suggests that MHC I may bind to cargo receptors, the identity of which remains elusive. Such a cargo was proposed to be Bap31, a transmembrane protein that cycles between ER and Golgi and interacts with both human and murine MHC I (49). However, knockdown of Bap31 does not lead to a decrease in MHC I expression at cell surface (50). Therefore, it has been suggested that additional and redundant mechanisms facilitate MHC I exit from the ER (51).

## Pathways of Cell-Surface MHC I Recycling in Non-Professional Antigen-Presenting Cells

Depending on the cell type, between 50 and 180% of plasma membrane surface is constitutively recycled by endocytosis (52). Membrane internalization by clathrin-mediated endocytosis is the best-characterized mechanism of endocytosis [for a review on clathrin-mediated endocytosis, the adaptors involved and the sorting signals recognized by the clathrin adaptors, see Ref. (53)]. Less well characterized, but also very important are the routes of clathrin-independent endocytosis, such as caveolae-mediated uptake, macropinocytosis, and constitutive clathrin-independent uptake [reviewed in Ref. (54, 55)]. These internalization routes permit endocytosis and recycling of plasma membrane proteins lacking any sorting signal for clathrin-mediated endocytosis, including the MHC I. Both fully conformed and empty MHC I are internalized via a clathrin- and dynamin-independent pathway. This pathway is ubiquitous, has been extensively studied in HeLa cells (56, 57), and requires free cholesterol and an active Arf6 GTP-binding protein.

The study of several mutant forms of Arf6 demonstrated that Arf6 is involved in both phosphoinositide kinase PI(4)P5K and phospholipase D activation (58–60). An Arf6-dependent formation of PIP_2_, the product of PI(4)P5K, might be required for the budding of tubular transport carriers directed to the plasma membrane (61), whereas the products of PLD activation could participate in a later fission step of such tubules in an analogous way to their proposed roles in the fission of COP-I transport carriers (62–64).

The majority of studies on Arf6-dependent endocytosis of MHC I analyzed the fully conformed MHC I recognized specifically by the conformational antibody W6.32. Recently, Zagorac et al. have investigated in parallel the endocytosis of fully conformed MHC I and empty MHC I (visualized by the non-conformational antibody HC-10), but without analyzing their colocalization with Arf6 (65). In this study, several alleles of fully conformed and empty MHC I were found in the same population of early endosomes. This suggests either that both forms of MHC I are internalized by the same route, or that early after their internalization they converge toward the sorting endosomes (SE) independently of how the initial internalization step at the plasma membrane took place. Considering that empty MHC I and conformational MHC I seemed to localize to distinct membrane domains at the cell surface, and that empty MHC I is internalized five to eight times faster than conformational MHC I (66), it is possible that the initial internalization step is different for empty and fully conformed MHC I.

The dichotomy between empty and conformational class I trafficking is obvious in later steps of endocytosis. In these steps, empty MHC I colocalizes with EGF receptor in late endosomes and is directed to lysosomes. In contrast, fully conformed MHC I remains longer in the early endocytic pathway, its localization overlaps with early endosomal antigen 1 and transferrin receptor (65), and reaches endosomal tubular structures similar to the Eps 15 homology domain protein 1 (EDH-1) recycling endosomes that require Arf6 activity (67). As a concluding remark of these studies, one might say that empty MHC I are rapidly internalized and at the level of SE are directed toward the lysosomes, while a part of conformational MHC I recycle back to plasma membrane by a tubular compartment, which requires Arf6, EDH-1 (67), and Rab22 and Rab11 (68, 69) [reviewed in Ref. (51)].

## A Potential Role of Ubiquitylation in MHC I Endocytosis

Viral immune evasion mechanisms often depend on viral proteins that interfere with MHC I trafficking. Well-known examples are MHC I retention in the ER by the US3 protein of HCMV (70), cytosolic MHC I degradation by the US11 protein of HCMV (71), MHC I endocytosis promoted by the Nef-1 protein of human immunodeficiency virus (HIV) (72) or by the K3 and K5 proteins of Kaposi sarcoma-associated herpes virus (73). The last example suggests that a potentially important event in MHC I recycling could be the ubiquitylation of lysine 63 of its HC cytosolic tail by the K3 and K5 ubiquitin ligases. Ubiquitin provides a signal for protein incorporation into intralumenal vesicles of multivesicular bodies (MVBs), a subpopulation of late endosomes that fuse with lysosomes (74). Thus, ubiquitylated MHC I is directed to lysosomes where it is degraded (75), allowing virus-infected cells to escape recognition by cytotoxic T cells. Beside the involvement in immune evasion mechanisms, regulation of MHC I by ubiquitin ligases might be a physiological phenomenon, since an endogenous ubiquitin ligase family similar to K3 and K5 proteins was discovered (76). This includes 11 mammalian membrane-associated RING-CH (MARCH) proteins that have been shown to increase the trafficking to late endosomes of several proteins internalized via clathrin-independent endocytosis (77). Among them, MARCH IV and MARCH IX over-expression leads to ubiquitylation and lysosomal degradation of MHC I (78), while MARCH I controls trafficking of MHC II during DCs maturation (79). The pattern of expression of MARCH IV and IX proteins in DC subpopulations, their transcriptional regulation, and their impact on both fully conformed and empty MHC I trafficking in DCs remain to be investigated and are of a great interest for the cellular biology of cross-presentation.

## Early Studies of MHC I Trafficking in DCs

**Figure 2:**
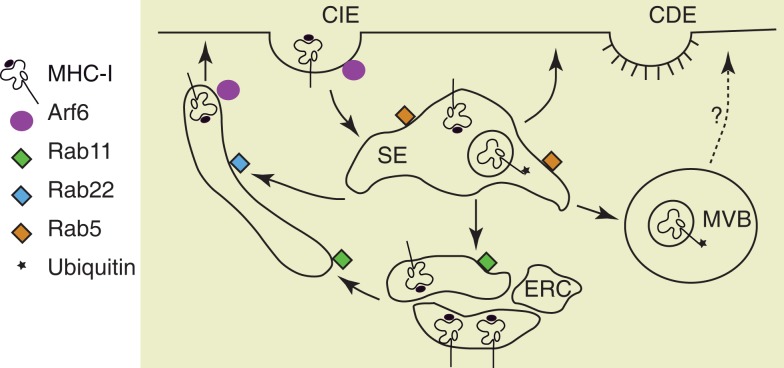
**Pathways of MHC I recycling**. Mammalian cells have two major pathways of endocytosis, clathrin-dependent endocytosis (CDE) and clathrin-independent endocytosis (CIE). MHC I molecules are internalized via CIE and arrive in sorting endosomes (SE). Depending on their conformation and their ubiquitination status, the MHC I are either recycled or directed to multivesicular bodies (MVB). The recycling of MHC I occurs via the endocytic recycling compartment (ERC) described by the small GTPase Rab11 or via tubular structures dependent on Rab22 and Arf6 (68, 69). Among these MHC I recycling pathways that were described in HeLa cells, only the MHC I from Rab11^+^ ERC was recently explored for their function in cross-presentation after antigen phagocytosis (118).

Almost everything that we know about endocytosis and recycling of MHC was learnt through the study of non-professional antigen-presenting cells, leading to the current overview of MHC I endocytosis depicted in Figure 2. However, a few studies on MHC I trafficking have been done in DCs, especially in monocytes-derived cells and DC-like cell lines. Early studies on murine DC-like cell line D1 (80) and human monocyte-derived DCs (81), showed that DCs activation by pathogens or TLR ligands increases MHC I biosynthesis and its stability at cell surface. Later studies discovered that MHC I intracellular distribution changes during DCs maturation. Thus, Delamarre et al., when analyzing MHC I localization in murine bone marrow-derived DCs (BM-DCs), demonstrated that in immature BM-DCs MHC I is intracellular, colocalizing with the Golgi marker GM130. Upon BM-DCs maturation by LPS and cluster disruption, the expression of MHC I at cell surface is up-regulated, being sevenfold higher than in immature BM-DCs (82). Like in BM-DCs, in human monocyte-derived DCs and in a DC-like cell line (KG-1), MHC I trafficking is regulated during DCs maturation (83). In immature human DCs, cell-surface MHC I has a short half-life at cell surface and about 60% of MHC I is sequestered in the proximal Golgi apparatus, colocalizing with GM130, γ-adaptin, and GRASP22. Upon DC activation by LPS, MHC I is relocated at cell surface, where it has an increased half-life, in correlation with down regulation of its endocytosis (83). The molecular adaptors that retain MHC I in Golgi remain still unknown, even if a possible role was commented for CD99, which has been reported to be required for MHC I anterograde transport from the ER in B cells (84). Different from monocyte-derived DCs, in immature human Langerhans cells (LCs), MHC I is stored in intracellular vesicles, partially overlapping with HLA-DM. LPS-induced maturation of LCs only modestly increases MHC I biosynthesis, but induces a rapid mobilization of the intracellular pool of MHC I to cell surface (85).

These initial studies on MHC I localization in human monocyte-derived DCs and LC indicate that the intracellular localization and trafficking regulation vary from one DC type to another. A careful and systematic examination of MHC I localization in several DC subpopulations has not been done yet. Nevertheless, this will be required in the future, to be able to estimate to what extent the molecular mechanisms regulating MHC I trafficking in monocyte-derived DCs, which will be discussed in subsequent sections of this review, operate in other DC subsets.

## Pathways of Antigen Processing during Cross-Presentation

Early studies on molecular mechanisms allowing exogenous antigens presentation on MHC I established that two major pathways of antigen processing exist during cross-presentation: one that is TAP and proteasome-dependent and another that is TAP and proteasome-independent. In the proteasome-independent cross-presentation, known as vacuolar cross-presentation, acidic lysosomal proteases, among which the Catepsin S seems to have a major role (86), generate the MHC I ligands in the endocytic pathway. Therefore, in proteasome-independent cross-presentation, MHC I loading occurs in endocytic vesicles. The vacuolar route of MHC I cross-presentation is considered to be less effective than proteasome-dependent cross-presentation (87). Nevertheless, from the data available today it is problematic to get definitive conclusions on the relative importance of each cross-presentation pathway because proteasome inhibition has pleiotropic cellular effects while TAP deficiency also alters MHC I trafficking.

Contrary to vacuolar cross-presentation in which the antigen processing occurs in the endocytic vesicles, TAP and proteasome-dependent cross-presentation involves transport of exogenous antigen from the endocytic pathway to the cytosol. The unique ability of DCs to export the antigenic material from the endocytic vesicles to the cytosol (88) is probably the key feature that allows them to excel in cross-presentation of exogenous antigens. Antigenic material shuttled into cytosol is digested by the proteasome, which is the main cytosolic broad substrate specificity protease. The proteasome generates precursors of antigenic peptides that are longer than 8–10 amino acids, which is the optimal peptide length for an MHC I ligand (89). Thus, the ligand precursors generated by the proteasome have rarely the optimal length and require further processing by accessory peptidases, either in the cytosol or in the ER [for a review, see Ref. (90)].

## Aminoterminal Trimming of Antigen Precursors and MHC I Loading Compartments during Cross-Presentation

The enzymes that perform the final step of antigen processing for MHC I antigen presentation belong to the same subfamily (IRAP subfamily) of M1 metallo-peptidases and are encoded by genes located in the same region of human chromosome 5. In humans, this family includes two ER localized enzymes, ER trimming aminopeptidases (ERAP)1 and ERAP2 (91), and the endosomal enzyme insulin-responsive aminopeptidase (IRAP) (92). Rodents have only two antigen trimming aminopeptidases, IRAP in endosomes, and the equivalent of human ERAP1, ERAAP in the ER (93). ERAPs are soluble, intraluminal proteins, while IRAP is a type II transmembrane protein that bears an intracytosolic domain of 110 amino acids. This cytosolic domain contains all the information required for IRAP trafficking, as demonstrated by extensive study of the enzyme in insulin-responsive tissues (94, 95), where a major cargo of IRAP vesicles is the insulin-regulated glucose transporter Glut4 (96). In DCs, IRAP is found in intracellular vesicles that contain also MHC I molecules, the small GTPase Rab14, and the Q-SNARE Syntaxin 6. These vesicles are recruited to early phagosomes and endosomes containing the internalized antigen and facilitate cross-presentation ability of DCs by antigen trimming function of IRAP (92, 97). Interestingly, IRAP not only colocalizes with internalized MHC I molecules, but also co-immunoprecipitates with MHC I. Why the endosomal trimming aminopeptidase interacts with MHC I is still unknown. The close proximity of IRAP and MHC I molecules might facilitate the loading of MHC I in endosomes and phagosomes, an environment that might be less favorable than the ER for class I loading. Alternatively, IRAP, which bears two di-leucine motifs in its cytosolic domain might interact with clathrin adaptors and direct the vesicular trafficking of MHC I molecules. To understand the functional role of IRAP interaction with MHC I, these hypotheses need further investigation.

**Figure 3:**
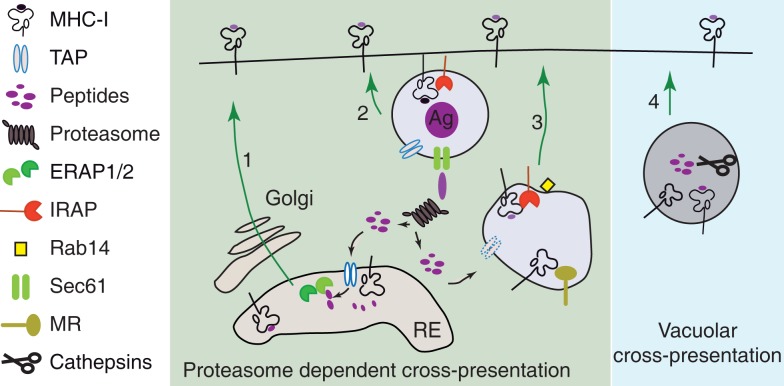
**MHC I loading compartments during cross-presentation**. (1) The antigen precursors produced in the cytosol of DCs can join the classical MHC I presentation pathway and can be transported by TAP in ER. In ER, the final trimming of antigen precursors is performed by the ER aminopeptidases (91, 93, 121, 122) and the resulting peptides bind to newly synthetized empty MHC I (92, 123, 124). (2 and 3) Proteasome digestion products generated in the cytosol of DCs can be retro-transported in phagosomes (111, 112, 114) or specialized endosomes (117, 125). The final step of antigen processing is probably done by IRAP which is the main trimming aminopeptidase recruited to the phagosome and colocalizing with mannose receptor endosomes (92). (4) Alternatively, in proteasome-independent cross-presentation (vacuolar pathway of cross-presentation), the exogenous antigen is entirely processed in the endocytic pathway by endolysosomal hydrolases (86).

The discovery and detailed characterization of the localization of trimming peptidases and their roles in cross-presentation allowed us to speculate on the cellular compartments in which MHC I is loaded with peptides (Figure 3). In the proteasome-dependent cross-presentation pathways, these compartments are the perinuclear ER, the phagosomes, and the specialized endosomes, while in the vacuolar pathway of cross-presentation the MHC I loading occurs in late endosomes or lysosomes.

## The Intracellular Origin of Cross-Presenting MHC I

Although, theoretically, MHC I loading with exogenous peptides during cross-presentation could take place in the perinuclear ER, several lines of evidence accumulated in the last decades that in, DCs, the main components of antigen processing machinery have access to antigen containing endosomes and phagosomes. This enhanced access is likely to contribute to a high efficiency of the cross-presentation phenomenon, as summarized in the Figure 3. The existence of peptide-receptive MHC I molecules is the primordial ingredient for cross-presentation. Thus, to understand the molecular mechanisms of cross-presentation, the origin of MHC I molecules that are loaded with exogenous peptides in the endocytic pathway is a central question investigated by several recent reports.

In the proteasome-independent vacuolar pathway of cross-presentation, initial studies clearly indicated that the MHC I that are loaded with antigenic peptides are recycling MHC I (98–101), even if the molecular mechanisms of MHC I endocytosis and recycling have not been investigated systematically in that work. However, the most recent exploration of MHC I intracellular localization during cross-presentation gave new insights into MHC I vesicular trafficking. Thus, in a model of viral infections as well as soluble ovalbumin cross-presentation, Lizée et al. demonstrated that a conserved tyrosine in the cytosolic tail of MHC I is required for MHC I endocytosis and its targeting to lysosomal vesicles (102). The lysosomal-targeted class I molecules were probably loaded with antigenic peptides generated in the endocytic pathway, although the proteasome dependence of the cross-presentation models used in these studies (102, 103) has not been investigated. The molecular mechanism by which the tyrosine residue in the cytosolic tail of MHC I favors its endocytosis and lysosomal targeting remains enigmatic and the cytosolic adaptor that recognizes this MHC I trafficking motif remains to be identified.

Additional evidence that the cytosolic tail of MHC I contains a cryptic sorting signal around the previously mentioned tyrosine residue comes from the study of molecular mechanisms by which the HIV down-regulates the cell-surface MHC I level (104–106). In the presence of HIV Nef protein, the cryptic signal YXXXAXXD of MHC I binds the AP-1 clathrin adaptor (107) and this trimolecular complex directs MHC I to endosomes and lysosomes. Interestingly, while in the presence of Nef protein, the cross-presentation by MHC I containing the tyrosine signal (HLA-A2, HLA-B) is compromised, both the clathrin adaptor AP-1 and the tyrosine motif of MHC I are absolutely required for cross-presentation of soluble ovalbumin (108). If these data clearly indicate a role for AP-1 in targeting MHC I from trans Golgi to intracellular vesicles prior to presentation at the cell surface, the nature of these intracellular vesicles nor the mechanism of AP-1 binding to MHC I in the absence of Nef is known. The example of HIV Nef protein that reorients MHC I trafficking at trans Golgi level via AP-1 clathrin adaptor might indicate the existence of an endogenous protein that can substitute for Nef protein and ensure AP-1 binding to MHC I. Such a candidate is the class II invariant chain Ii, which binds AP-1 clathrin adaptor (109) and which has been shown to be required for MHC I sorting to endosomes and lysosomes (110).

The majority of data supporting the role of MHC I cytosolic tail in MHC I trafficking and cross-presentation were obtained in cross-presentation settings that often used soluble antigens and were possibly proteasome-independent. To what extent these findings apply to phagosome-mediated and receptor-targeted cross-presentation, which are usually proteasome-dependent, remains to be elucidated.

Contrary to vacuolar cross-presentation, in the proteasome-dependent cross-presentation, the source of MHC I molecules that are loaded with exogenous peptides has been considered for a long time to be the MHC I from the ER. This assumption is also compatible with the fusion of ER with phagosomal membranes, which provide to phagosomes several components of MHC I antigenic presenting machinery, including MHC I molecules (111–113). It is likely that ER-phagosome fusion results in a mixed population of MHC I molecules, since both Endo H sensitive (ER form) and Endo H resistant (surface form) MHC I can be detected in phagosomes [(114) and our unpublished results]. Several recent data allow us to reconsider this original hypothesis and seem to indicate that the source of MHC I involved in proteasome dependent cross-presentation is not the newly synthetized class I from the ER. Thus, we have showed that preincubation of TAP-deficient DCs at 26°C increases the cell-surface level of MHC I and fully restores the cross-presentation of phagocytized antigens, in a model of proteasome-dependent cross-presentation (115). These experiments suggest that MHC I molecules from the late steps of secretory pathways or from the cell surface are essential in proteasome-dependent cross-presentation of phagosomal antigens. Interestingly, in the same study, the recovery of cell-surface MHC I by low temperature incubation of TAP-deficient DCs did not restore the cross-presentation of antigen targeted ovalbumin, indicating either that the source of loadable MHC I are different between phagocytized and receptor-targeted antigens, or that the phagosomes, but not the endosomes, are equipped with an alternative mechanism for peptide import, such as Sec61 (114).

Further proof in favor of the involvement of the recycling MHC I in proteasome-dependent cross-presentation was provided by a recent study performed in the DC-like cell line DC2.4, in which the cross-presentation of *E. Coli* expressing ovalbumin was investigated after siRNA-mediated inactivation of 57 Rab proteins (116). Several Rabs were required for optimal cross-presentation, such as Rab3b, 3c, 5b, 8b, 10, 33a, 34, and 35. Among them, Rab3 seems to be required for recycling of cell-surface MHC I, as demonstrated using ZsGreen β_2_m as a tracker. Additional strong evidence against the implication of ER localized MHC I molecules in cross-presentation of phagosomal antigens result from a very recent study on Rab11 role in MHC I trafficking during cross-presentation that will be presented in the next paragraphs.

## Control of MHC I Antigen Cross-Presentation by TLR Signaling

**Figure 4:**
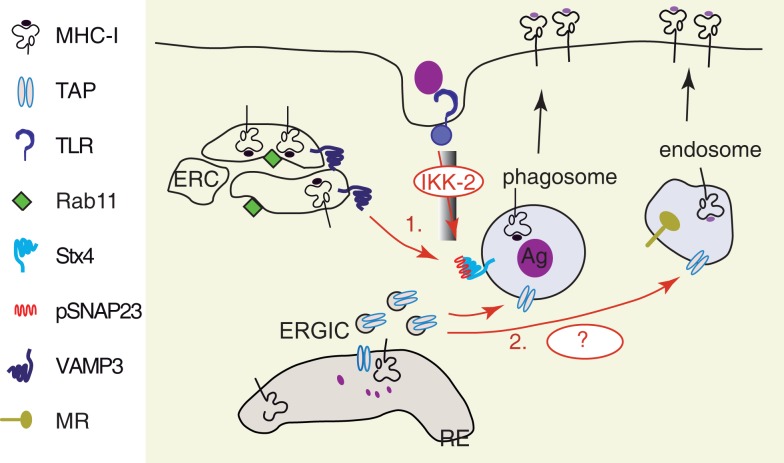
**TLRs activation facilitates the recruitment of MHC I and TAP to antigen containing vesicles**. (1) Following TLR activation, the downstream signaling cascade involving MyD88 drives phosphorylation of SNAP23 by IkB-Kinase (IKK2). Phosphorylated SNAP23 facilitates MHC I recruitment from Rab11^+^ ERC to phagosomal membrane, probably by stabilization of SNARE complexes (118). (2) Sec22b-mediated ER–phagosome fusion provides TAP to the phagosomal membrane in the absence of TLR activation (113). A further increase in TAP recruitment to antigen containing vesicles, such as MR^+^ endosomes (117) and yeast containing phagosomes (92), has been observed after TLR stimulation. The underlying molecular mechanisms remain to be investigated.

The well-known ability of cross-presentation to induce both T cell priming and T cell tolerance is associated to activation of PRRs (29), among which the most potent and the best studied are the TLRs. The increase in co-stimulatory molecules and pro-inflammatory cytokine production by DCs enhance the potential of TLR-activated DCs to cross-prime the naïve CD8^+^ T cells. In addition to these effects of TLR activation, it has been recently demonstrated that the intracellular trafficking of two essential components of MHC I antigen-presentation machinery, TAP, and MHC I is regulated by TLR signaling (Figure 4). In a model of mannose receptor (MR) targeted cross-presentation, Burgdorf et al. demonstrated that TAP is enriched in MR endosomes upon DCs stimulation by a TLR4 ligand, the LPS (117). In a model of cross-presentation upon phagocytosis of yeast cells expressing ovalbumin, we detected a moderate increase in the amount of TAP recruited on phagosomes containing yeast cells when compared with the phagosomes containing latex beads (92). More recently, it has been shown that Sec22b-mediated fusion of ERGIC derived vesicles to the phagosome even in the absence of TLR ligands on the phagosomal cargo (113), suggesting that the recruitment of TAP to phagosomal membrane is less dependent on TLR stimulation than the recruitment of MHC I.

The supply of MHC I molecules to the phagosomes is strongly enhanced by TLR stimulation (118). Nair-Gupta and colleagues demonstrated that upon TLR4 stimulation by LPS, the downstream signaling cascade induces the IKK2-dependent phosphorylation of synaptosome-associated protein of 23 kDa (SNAP 23) on the phagosomes containing the TLR ligand. The phosphorylated SNAP23/Syntaxin 4 (STX4) complex recruits the t-SNARE VAMP3 and this molecular complex mediates the membrane fusion between endosomes containing MHC I and phagosomes. The VAMP3 endosomal compartment that contains MHC I is described by the small GTPase Rab11a and is identified as the endosomal recycling compartment (ERC). How MHC I molecules are stored in DCs in the VAMP3^+^ Rab11^+^ ERC and if they are directly targeted from the transGolgi network to ERC or if they are internalized from the cell membrane is still unknown. The importance of tyrosine in the cryptic sorting motif of the cytosolic tail of MHC I and the role of AP-1 clathrin adaptor in MHC I targeting to VAMP3^+^ Rab11a^+^ ERC remain to be investigated as well. Finally, since SNAP 23 has the ability to interact not only with STX4 and VAMP3, but also with several others SNAREs (STX2, 6, 11, VAMP2, 8), the activation of SNAP 23 by TLR signaling might trigger important changes in vesicular trafficking in DCs. Thus, this regulation of membrane fusion machinery by TLR signaling might be an important checkpoint for antigen presentation and immunogenic capacities of DCs.

## Concluding Remarks

Cross-presentation of exogenous antigens via MHC I is a complex phenomenon, which is largely based on the high plasticity of the endocytic system in DCs. The main ingredient for successful cross-presentation is the presence of peptide loadable MHC I in the cellular compartment where the antigenic peptide is produced. While the pathways of intracellular trafficking of MHC I have been fairly well studied in model cell lines, such as HeLa cells, the study of MHC I trafficking in DCs is still in a pioneer stage and the few cellular biology studies focused on mouse BM-DCs. However, we think that the time has come to develop this topic since it could fully benefit from the comprehensive characterization of DC subpopulations and their equivalence between humans and rodents (23). In combination with the availability of high-resolution microscopy and excellent conformation-specific antibodies against human MHC I (119, 120), the characterization of MHC I trafficking in human DC subpopulations became possible.

Considering the well-known ability of signaling through PRRs to increase the immunogenic abilities of DCs, including cross-presentation (29) it is of outstanding interest to determine how TLR signaling regulates MHC I trafficking in DC subpopulations. The recent work of Nair-Gupta et al. has provided some insights on MHC I recruitment from Rab11^+^ ERC to antigen-containing phagosomes in mouse BM-DCs upon TLR2 and TLR4 activation via the MyD88 signaling pathway (118). To what degree these interesting findings apply to steady-state DC subpopulations or to accessory cross-presenting cells? Are Rab11^+^ ERCs a TLR-regulated reservoir of MHC I in all cells capable of cross-presentation? Is the TRIF-dependent signaling pathway able to regulate MHC I trafficking? Answering these questions in the near future would pave the way to a mechanistic comprehension of MHC I cross-presentation regulation through modulation of intracellular MHC I trafficking.

## Conflict of Interest Statement

The authors declare that the research was conducted in the absence of any commercial or financial relationships that could be construed as a potential conflict of interest.
